# Short-term outcomes in minimally invasive versus open gastrectomy: the differences between East and West. A systematic review of the literature

**DOI:** 10.1007/s10120-017-0747-0

**Published:** 2017-07-20

**Authors:** Nicole van der Wielen, Jennifer Straatman, Miguel A. Cuesta, Freek Daams, Donald L. van der Peet

**Affiliations:** Department of Gastrointestinal Surgery, VU University Medical Center, De Boelelaan 1117, ZH 7F020, 1081 HV Amsterdam, The Netherlands

**Keywords:** Minimally invasive, Gastric cancer, Gastrectomy

## Abstract

**Objective:**

Minimally invasive surgical techniques for gastric cancer are gaining more interest worldwide. Several Asian studies have proven the benefits of minimally invasive techniques over the open techniques. Nevertheless, implementation of this technique in Western countries is gradual. The aim of this systematic review is to give insight in the differences in outcomes and patient characteristics in Asian countries in comparison to Western countries.

**Methodology:**

An extensive systematic search was conducted using the Medline, Embase, and Cochrane databases. Analysis of the outcomes was performed regarding operative results, postoperative recovery, complications, mortality, lymph node yield, radicality of the resected specimen, and survival. A total of 12 Asian and 8 Western studies were included.

**Results:**

Minimally invasive gastrectomy shows faster postoperative recovery, fewer complications, and similar outcomes regarding mortality in both the Eastern and Western studies. However, patient characteristics such as age and BMI differ between these populations. Comparison of overall outcomes in minimally invasive and open procedures between East and West showed differences in complications, mortality, and number of resected lymph nodes in favor of the Asian population.

**Conclusion:**

Improved outcomes are observed following minimally invasive gastrectomy in comparison to open procedures in both Western and Asian studies. There are differences in patient characteristics between the Western and Asian populations. Overall outcomes seem to be in favor of the Asian population. These differences may fade with centralization of care for gastric cancer patients in the West and increasing surgical experience.

## Introduction

Based on the Japanese gastric cancer treatment guidelines, curative treatment for gastric cancer consists of surgical resection with adequate lymph node dissection. An adequate resection margin of 2 cm is required with T1 tumors, 3 cm for T2 tumors with an expansive growth pattern, and 5 cm for those with infiltrative growth patterns. Additionally, D1 or D1+ lymphadenectomy is indicated for cT1N0 tumors, and D2 is indicated for cN+ or cT2–T4 tumors [1]. Lymph node yield in gastric cancer surgery is strongly associated with survival and therefore considered a marker for quality of care [2–4]. Since Kitano et al. described the first laparoscopic distal gastrectomy in 1994, the minimally invasive technique has gained increased interest worldwide [5].

Most studies regarding minimally invasive gastrectomy showed good outcomes in countries such as Japan and Korea where there is a relatively high incidence of early gastric cancer because of the screening programs for gastric cancer [6, 7]. There is no screening program for gastric cancer in Western countries and a higher incidence of advanced gastric cancer occurs; thus, the results from Asian studies might not be directly applicable for Western countries. Controversy exists whether postoperative outcomes, radicality, and lymph node yield are influenced by the surgeon’s learning curve, resulting in a slow acceptance and implementation of minimally invasive gastrectomy in the Western world [8].

To compare the outcomes of minimally invasive surgical techniques with open surgical techniques for gastric cancer, several meta-analyses have been conducted in recent years [9, 10].

However, those studies did not differentiate between outcomes in Eastern Asia and the Western world. The aim of this study is to compare the outcomes of the studies conducted in Asia with the studies conducted in the West.

## Materials and methods

### Literature search

To identify all relevant publications, a systematic search was performed in the bibliographic databases PubMed, EMBASE.com, and The Cochrane Library (via Wiley) from inception to 24 January 2017. Search terms included controlled terms from MeSH in PubMed, EMtree in EMBASE.com, as well as free text terms. Free text terms were only used in The Cochrane library. Search terms expressing ‘stomach neoplasms’ were used in combination with search terms constituting ‘open surgery’ and ‘laparoscopy.’ The reference list of included articles was hand searched for relevant publications.

### Selection criteria

The search findings were independently evaluated for potential eligibility for this meta-analysis by two authors (J.S. and N.W.). The inclusion criteria were (1) the article had to compare minimally invasive gastrectomy with open gastrectomy; (2) only full text articles were included; case reports were not included; (3) the article had to be in English (no other language was accepted); and (4) only gastrectomy for gastric cancer was included. After this selection, another selection based on type of gastrectomy was made. In this systematic review only articles with total or total and subtotal gastrectomies combined were included.

### Study characteristics

The Newcastle-Ottawa Quality Assessment Scale (NOS) for retrospective cohort studies and case–control studies was used to assess the quality of the studies. A maximum of nine points could be awarded: four points for selection criteria, two points for comparability, and three points for outcomes. Studies achieving six or more points would be classified as high quality and were used for further analysis. Quality of randomized controlled trials (RCT) was assessed using the Jadad scale for RCT. A maximum of five points could be awarded: two points for adequate randomization, two points for adequate blinding, and one point if all included patients were accounted for.

### Definitions

The following definitions were used for the recorded parameters. Regarding operative data: operation duration was defined in minutes (min) and blood loss in milliliters (ml). Hospital stay: time to first flatus and time to first oral intake were reported in days. Definitions of complications varied between different studies: there was no consensus in reporting type or grade of complication such as the Clavien–Dindo grading system for the classification of surgical complications. Therefore, only the frequency of postoperative complications was reported. In-hospital mortality was defined as mortality during hospital stay or within 30 days postoperatively. Proximal and distal resection margins were reported in centimeters.

### Statistical analysis

The systematic review was performed in line with the recommendations from the PRISMA statement for reporting systematic reviews and meta-analyses [11]. Review Manager version 5.3.5 (2014) was used for data analyses. Continuous variables were assessed using the weighted mean difference. Dichotomous variables were assessed using the odds ratio. To account for clinical heterogeneity, the random effects model based on DerSimonian and Laird’s method was used [12]. A *P* value <0.05 was considered statistically significant.

A sub-analysis was made in Review Manager for studies conducted in Asian countries and studies conducted in Western countries. The analyses, performed using Review Manager, only compared the outcomes in the minimally invasive group with the outcomes in the open group. Therefore, calculation of the weighted means per outcome in the Western group and the Eastern group was conducted. Overall weighed means are displayed in Table 1.

**Table 1:** Overall weighted mean outcomes

	West		East	
	MIG	OG	MIG	OG
Age	67.54	68.74	60.70	59.44
BMI	23.97	24.38	22.35	22.69
Blood loss	149.64	397.48	83.52	213.05
Operative time	243.02	233.87	218.81	217.80
First diet	4.41	6.35	4.79	4.85
First flatus	2.90	5.75	3.93	3.96
Hospital stay	9.74	11.22	13.66	14.85
Complications (%)	21.69	30.80	12.23	15.79
Mortality (%)	3.27	5.81	0.33	0.24
Lymph nodes	22.96	18.88	31.29	32.35

*MIG* minimally invasive gastrectomy, *OG* open gastrectomy, *BMI* body mass index

## Results

### Study selection

The initial literature search resulted in 2182 hits. After deleting duplicate articles, 1429 articles remained suitable for analysis. After selection on title and abstract, 181 articles remained that met the criteria. Sixty articles did not meet the criteria and were not suitable for analysis after reading the full text. Thus, 121 suitable articles were left. After assessing type of gastrectomy and the quality of the study, using the Newcastle Ottawa quality assessment scale for cohort studies and the Jadad scale for randomized controlled trials, a total of 20 studies were included, 19 retrospective studies and 1 randomized controlled trial (RCT) [13–32]. Twelve studies were conducted in Asian countries; 8 studies were conducted in Western countries. An overview of the selected articles and patient characteristics is depicted in Tables 2 and 3.

**Table 2:** Newcastle Ottawa Quality Assessment Scale and Jadad score

References	Representatives of the exposed cohort	Selection of the nonexposed cohort	Ascertainment of exposure	Demonstration that outcome of interest was not present at start of study	Comparability of cohorts (max. 2 points)	Assessment of outcome	Follow-up long enough for outcome of interest	Adequacy of follow-up	Total
Cianchi [25]	1	1	1	1	2	1	1	1	9
Dulucq [26]	1	0	1	1	2	1	1	1	8
Ecker [27]	1	1	1	1	2	1	0	0	7
Guzman [28]	1	0	1	1	1	1	1	1	7
Pugliese [29]	1	0	1	1	1	1	1	1	7
Ramagem [30]	1	1	1	1	1	1	0	0	6
Siani [31]	0	1	0	1	2	1	1	1	7
Topal [32]	1	1	1	1	1	1	1	1	8
An [13]	1	1	1	1	2	1	1	1	9
Du [14]	0	1	1	1	2	0	1	1	7
Jeong [15]	1	1	1	1	1	1	1	1	8
Kawamura [16]	0	1	1	1	2	1	0	0	6
Kim [17]	0	1	1	1	2	0	1	1	7
Kim [18]	1	1	1	1	2	1	1	1	9
Lin [19]	1	1	1	1	1	1	1	1	8
Mochiki [20]	0	1	1	1	1	0	1	1	6
Sakuramoto [21]	0	1	1	1	1	0	1	1	6
Son [22]	0	1	1	1	2	1	1	1	8
Usui [23]	0	0	1	1	2	0	1	1	6

References	Randomization mentioned	Randomization appropriate	Blinding mentioned	Blinding appropriate	All patients accounted for	Total
Cui [24]	1	1	1	1	1	5

**Table 3:** Patient characteristics

References	Study period	Design	Country	Sample size		Mean age		Sex M/F		BMI		Tumor stage (%)				Lymph node dissection
				MIG	OG	MIG	OG	MIG	OG	MIG	OG	I	II	III	IV	
Cianchi [25]	06/2008 01/2012	Retrospective	Italy	41	41			24/17	25/16			28	18	54	0	D1+ α/β/D2
Dulucq [26]	04/1995 03/2004	Retrospective	France	8	11	75 ± 8	67 ± 14	3/5	5/6							D1+ β
Ecker [27]	01/1998 12/2011	Retrospective	USA	331	2303	68.85 ± 12.11	69.08 ± 13.01	204/127	1342/961			14.8	37.1	47.4	0	
Guzman [28]	11/1999 01/2009	Retrospective	USA	30	48	70	67	17/13	31/17	24.7	25.6	44.9	33.3	10.3	11.5	D1/D2
Pugliese [29]	06/2000 06/2005	Retrospective	Italy	48	99							85.4	6.3	8.3	0	D1+/D2
Ramagem [30]	08/2009 04/2013	Retrospective	Brazil	47	64	57.8 ± 10.53	59.7 ± 11.65	34/13	43/21	23.2 ± 4.36	23.8 ± 4.04	31.5	26.1	42.4	0	D2
Siani [31]	01/2003 10/2009	Matched cohort	Italy	25	25	65 ± 8.5	66 ± 7.8	15/10	18/7			20	20	60	0	
Topal [32]	01/2003 12/2006	Retrospective	Belgium	38	22	66.3 ± 12.3	66.9 ± 14.6	23/15	17/5	24.3 ± 2.8	23.4 ± 4.1	40	23.3	26.7	10	D2
An [13]	2003–2007	Retrospective	Korea	42	162	57.0 ± 11.6	56.6 ± 12.0	23/19	108/54							D1+ β/D2
Du [14]	11/2005 05/2009	Retrospective	China	82	94	60.4 ± 18.5	57.8 ± 17.2	54/28	61/33	22.3 ± 2.6	22.5 ± 2.4	5.1	38.1	56.8	0	D2
Cui [24]	10/2010 09/2012	RCT	China	128	142	60.1 ± 12.6	57.5 ± 11.2	88/40	98/44	23.03 ± 3.61	23.66 ± 3.23	21.8	26.7	51.5	0	D2
Jeong [15]	01/2005 12/2007	Retrospective	Korea	261	137							69.3	12.8	13.6	4.3	
Kawamura [16]	01/2003 12/2008	Retrospective	Japan	42	30	63.6 ± 10	64.9 ± 10.5	32/10	21/9	22.7 ± 3.1	22.7 ± 2.9	100	0	0	0	D2
Kim [17]	01/2004 07/2006	Retrospective	Korea	27	33	57.3 ± 14.2	61.6 ± 9.2	16/11	23/10	22.6 ± 3.1	22.4 ± 2.1					D1+ α/β/D2
Kim [18]	01/2009 04/2010	Retrospective	Korea	63	127	55.9 ± 12.2	57.3 ± 11.1	43/20	81/46	22.7 ± 2.5	23.0 ± 2.9					D2
Lin [19]	01/2005 10/2013	Retrospective	China	2041	1539	61.0 ± 11.1	59.8 ± 10.8	1523/518	1170/369	22.3 ± 3.2	22.6 ± 3.5	26.6	19.9	53.6	0	D1+ α/β/D2
Mochiki [20]	04/1998 12/2007	Retrospective	Japan	20	18	66 ± 2.4	63 ± 2.2	16/4	16/2			92.1	2.6	5.3	0	D1+ β
Sakuramoto [21]	07/2003 07/2007	Retrospective	Japan	30	44	63.7 ± 9.2	67.2 ± 9.9	12/18	10/34	21.9 ± 2.7	22.5 ± 3.6	54	25.7	20.3	0	D1+ β/D2
Son [22]	05/2003 12/2009	Retrospective	Korea	39	22	50.7 ± 14	52.4 ± 12.9	13/26	10/12			0	32.8	67.2	0	D1+/D2
Usui [23]	05/2011 08/2004	Retrospective	Japan	20	19	66.0 ± 10.4	66.2 ± 10.2	13/7	14/5	21.3 ± 3.1	22.1 ± 2.4	92.3	7.7	0	0	

### Operative results

Operation duration was shorter in the open group in both the Western studies and the Asian studies. The overall results of both subgroups showed a significant difference. Overall weighted mean difference was 30.84 min (95% CI, 8.12–64.81).

**Fig. 1:**
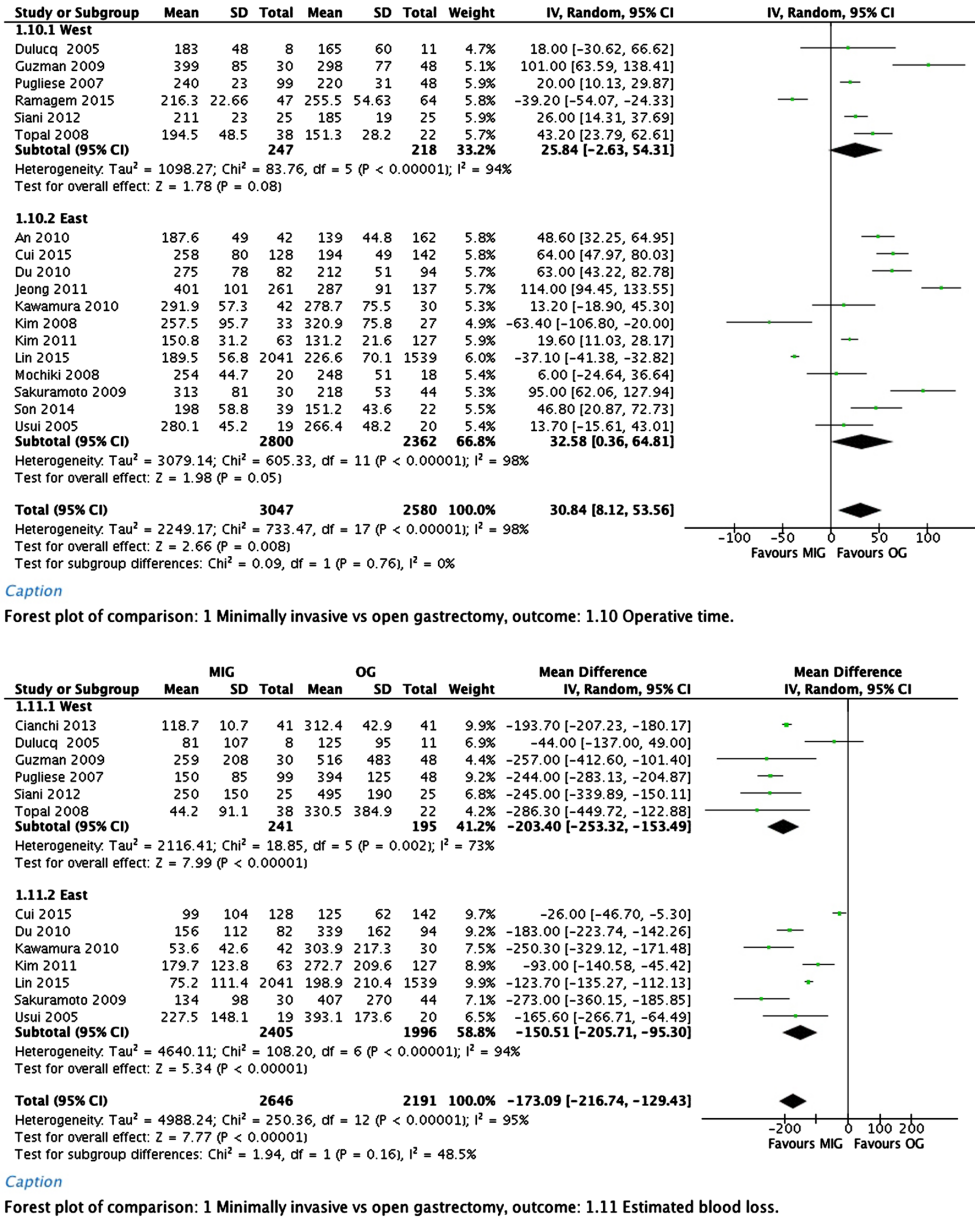
Operation duration and blood loss

Blood loss was significantly less in the minimally invasive group. Overall weighted mean difference was −173.09 ml (95% CI, −216.74 to −129.43). Forest plots of operation duration and blood loss are depicted in Fig. 1.

### Postoperative recovery

Hospital stay was significantly shorter in the minimally invasive group. Overall weighted mean difference was −3.46 days (95% CI, −4.49 to −1.63).

The time to first flatus showed a significant difference in favor of the minimally invasive group. In the analysis of only the Asian studies this was not significantly different; the overall weighted mean difference was −1.08 days (95% CI, −7.97 to −0.19).

Time to first diet showed a significant difference in favor of the minimally invasive group. Overall weighted mean difference was −1.85 days (95% CI, −3.61 to −0.10).

**Fig. 2:**
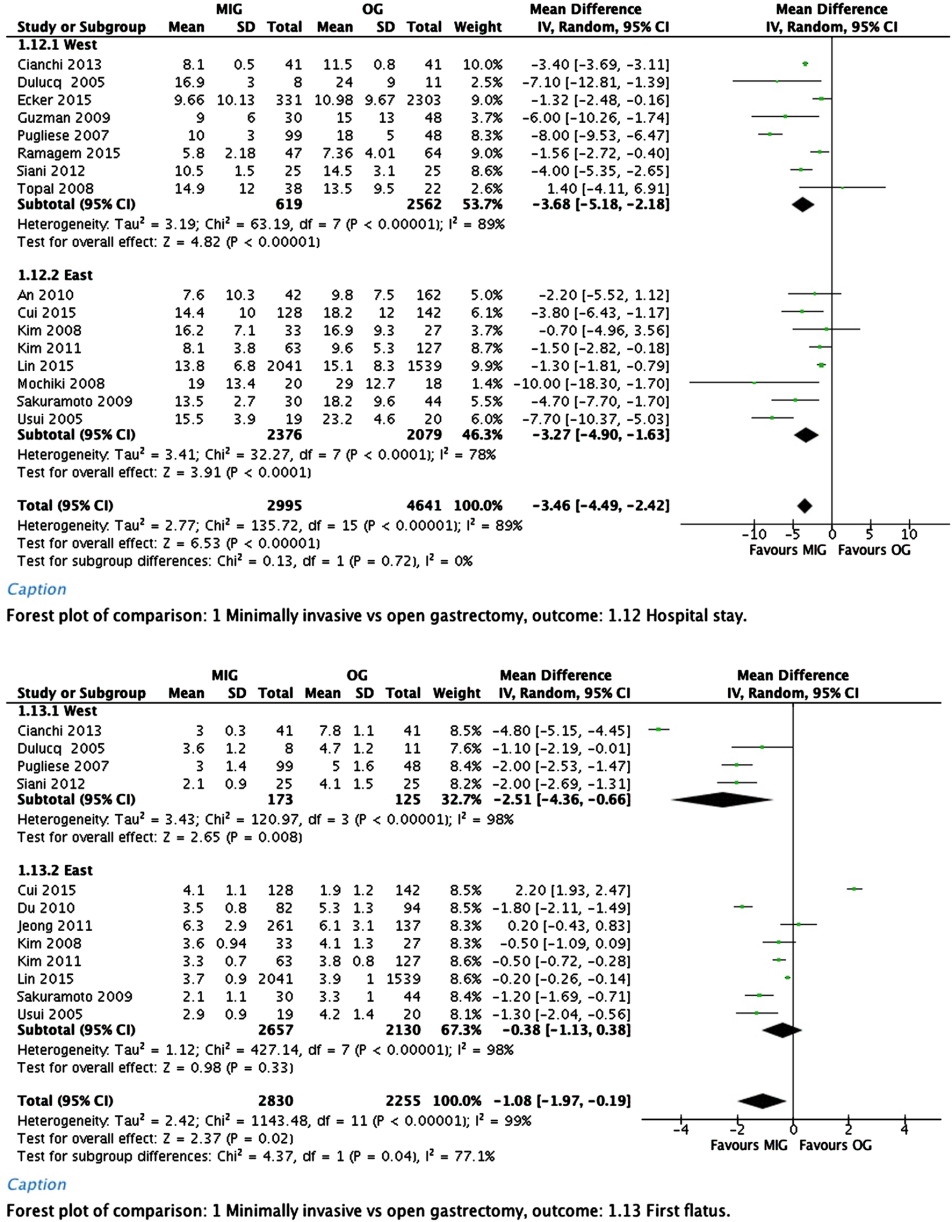

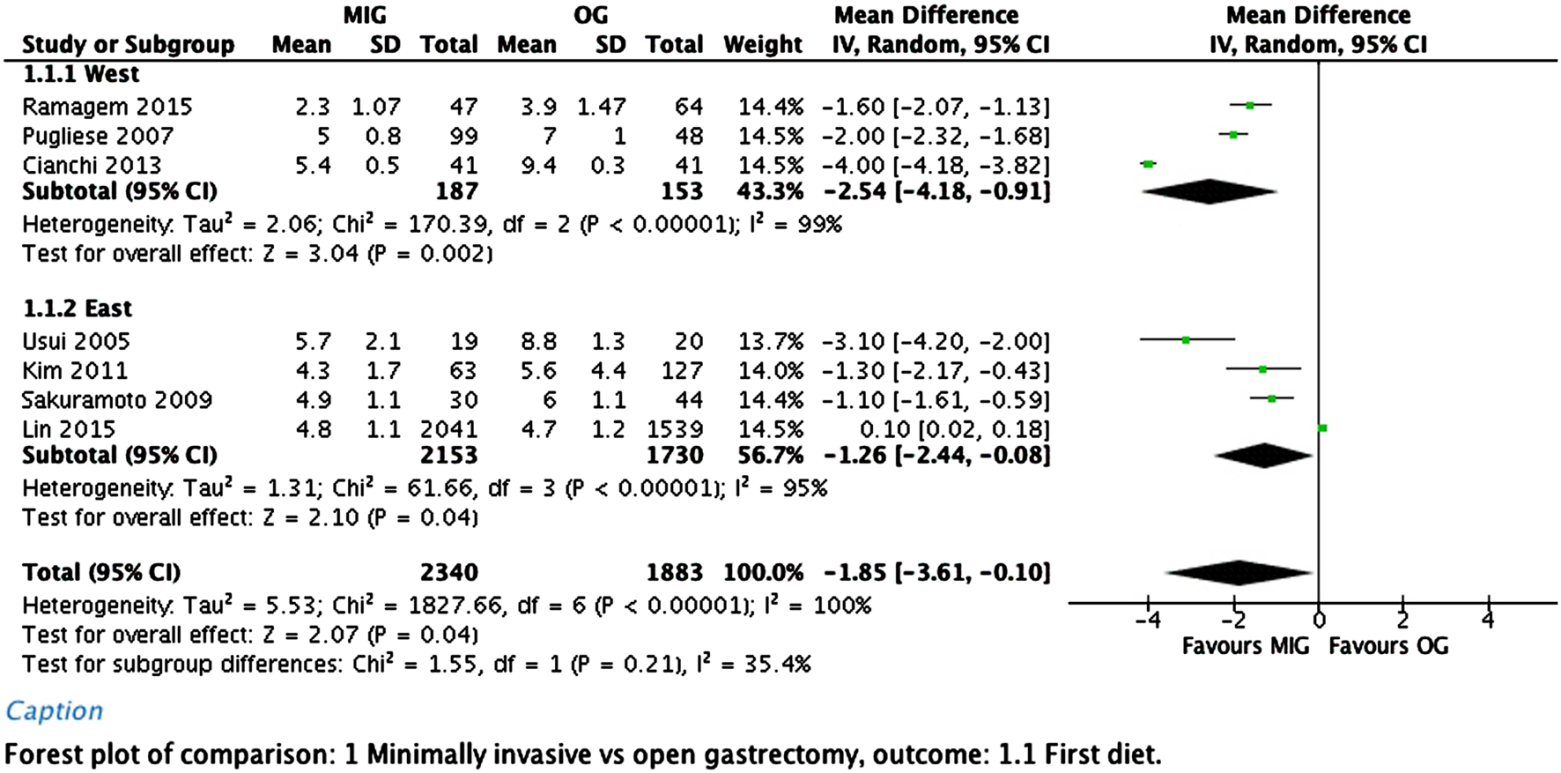
Hospital stay, time to first flatus, and time to first diet

Forest plots of postoperative recovery are depicted in Fig. 2.

### Morbidity and mortality

Fewer complications occurred in the minimally invasive group in comparison to the open group. Analysis of only the Asian studies showed no significant difference between the two groups. However, overall the analysis showed a significant difference with an odds ratio (OR) of 0.73 (95% CI, 0.58–0.92).

**Fig. 3:**
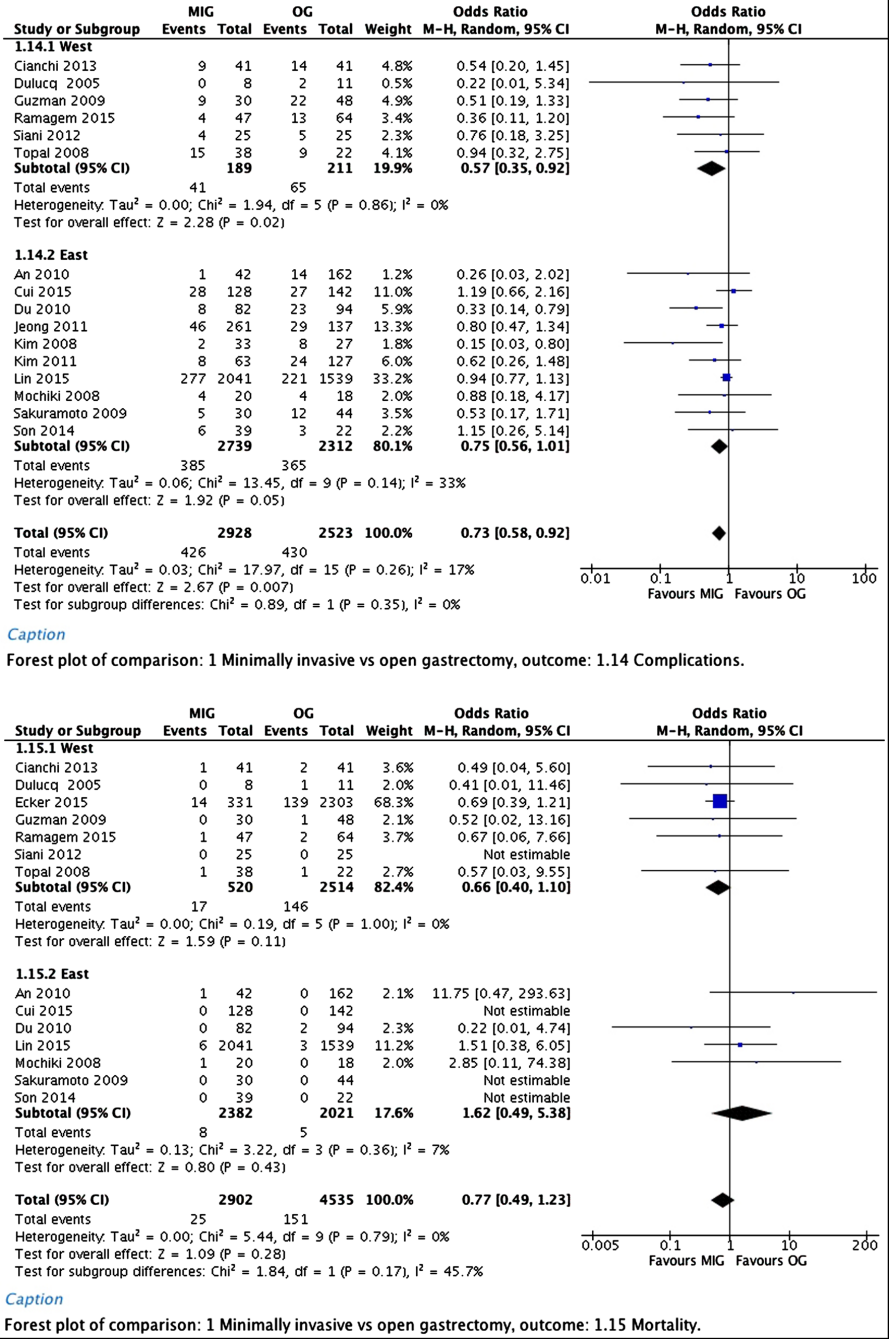
Postoperative complications and mortality

No difference in mortality was seen between the two groups, with an odds ratio of 0.77 (95% CI, 0.49–1.23). Forest plots of morbidity and mortality are depicted in Fig. 3.

### Oncological outcomes

**Fig. 4:**
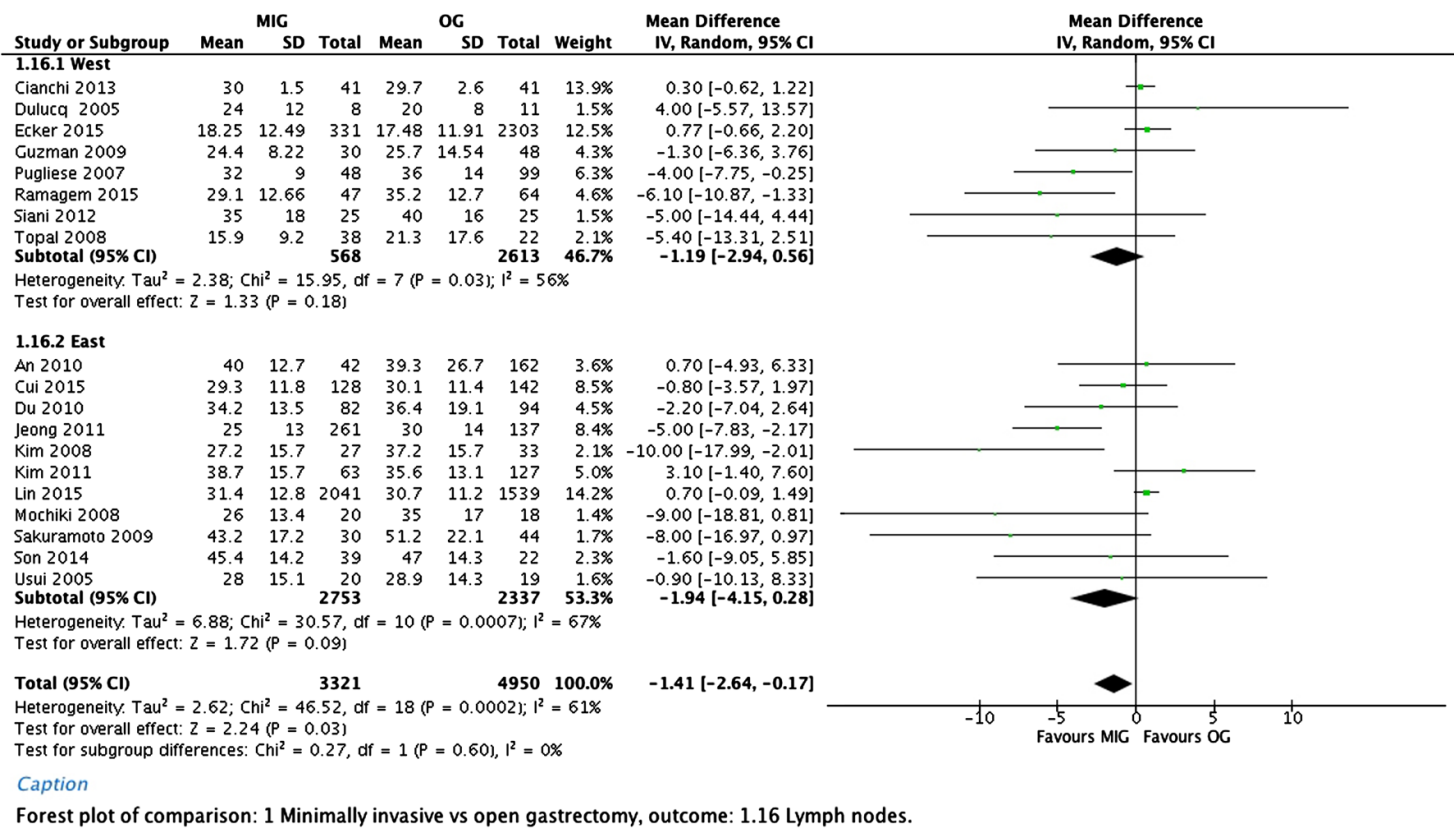
Lymph node yield

Significantly more lymph nodes were resected in the minimally invasive group in comparison to the open group, with an overall weighted mean difference of −1.41 lymph nodes (95% CI, −2.64 to −0.17). A forest plot of lymph node yield is depicted in Fig. 4.

Four Asian studies reported radicality; all reported 100% R0 resections [13–15, 22, 24]. Seven Western studies reported radicality [25–30, 32]. Only one had all R0 resections, all other studies also reported R1 resections; however there were no differences between the open or minimally invasive groups.

Seven Asian studies reported disease-free and/or overall survival [13–15, 19–22]. Three studies conducted in Western countries reported disease-free and/or overall survival [26, 29, 31].

Follow-up duration differed between studies, making it difficult to compare these results. Only one study, that by Lin et al., reported a significant difference in survival between the two groups. In the overall 3-year disease free survival, a better survival rate was reported for the minimally invasive group. After comparing survival rates according to tumor stage, this difference disappeared.

## Discussion

In conclusion, improved outcomes are observed following minimally invasive gastrectomy in comparison to open procedures in both Western and Asian studies.

There are differences in patient characteristics between the Western and Asian populations. Patient characteristics such as age and body mass index (BMI) are higher in the Western population, which may be explained by the fact that the incidence of gastric cancer is higher in Eastern Asia. Several Asian countries have a screening program for gastric cancer. In Japan, population-based screening is recommended for individuals older than 50 years and in Korea for individuals aged 40–75 years, resulting in a lower age of onset compared to the West [7]. A higher BMI in the Western patient group could correspond to an overall higher BMI in the Western population. In the Netherlands more than 50% of the adult population has a BMI of 25 or higher, and approximately 36% had a BMI between 25 and 30 [33]. Mean BMI in gastric cancer patients in 2014 was 25 [34]. With more-advanced disease in gastric cancer in the West, an average normal BMI might reflect a cachectic overweight patient.

Both Western and Asian studies show better short-term outcomes in favor of minimally invasive surgical techniques, with faster postoperative recovery with a significant shorter hospital stay, shorter time to first flatus, and a shorter time to first diet. It should be noted that international implementation of enhanced recovery protocols (ERAS) might influence these results. Perioperative results show significantly less blood loss in the minimally invasive group; however, operation duration was longer. Additionally, fewer postoperative complications and no differences in mortality were reported. All these short-term advantages can be attributed to the less invasive nature of the minimally invasive approach. These outcomes are in accordance with other meta-analyses comparing minimally invasive with open gastrectomies [9, 10].

When comparing East with West, more blood loss and longer operation duration are seen in the Western studies, which may be attributed to the lower overall incidence of gastric cancer in Western countries. Blood loss and operation duration might not have clinical value, but these outcomes could indicate Western surgeons have less experience in the treatment of gastric cancer. Only one study reported on surgeon experience; one experienced surgeon performed all minimally invasive procedures [32]. It should be noted that the incidence of performing minimally invasive gastrectomy was low in the Western studies. In all but one Western study fewer than 10 minimally invasive procedures were performed annually. With a learning curve reported at 20–40 procedures, this finding indicates progression through the learning curve is slow and might affect the presented results [35].

With regard to complications and mortality, these outcomes seem to show a trend in favor of the Asian studies. There are two large studies reporting the outcomes of mortality rate [19, 27]. Analyses with and without these studies did not show an effect on the outcomes presented here. An overall significant difference in lymph node yield in the minimally invasive group was seen, with a mean difference of 1.41 lymph nodes. No effects on clinical outcome are to be expected from this result. More importantly, all studies reported an average of at least 19 resected lymph nodes, which is in accordance with the recommendations of the Japanese Gastric Cancer Association, which advocates removal of at least 15 lymph nodes [1]. The overall differences in lymph node yield show a trend toward a higher number of resected lymph nodes in the Eastern studies, which can be attributed to the greater experience of Asian surgeons with the surgical treatment of gastric cancer. Additionally, pathological examination of lymph nodes can be different, with lymph node dissection taking place separately or en bloc. Furthermore, examination by a specialized upper gastrointestinal pathologist could influence the outcome. Unfortunately, no study reported if lymph node yield was done separately or en bloc.

The difference in survival reported by Lin et al. could be explained by the heterogeneity of both groups, with larger tumors and more advanced disease in the open group [19].

Overall outcomes seem to be in favor of the Asian population. Future research should aim to further assess differences in population, patient assessment, surgical techniques, and experience, to ensure optimal treatment for all gastric cancer patients. In the West care for gastric cancer patients is more and more centralized to specialized treatment units, ensuring optimal care, not only by experienced surgeons, but by an experienced treatment team, ranging from preoperative workup to perioperative care and follow-up. The implementation of minimally invasive techniques for gastric cancer is progressing gradually in Western countries. Several randomized controlled trials are being conducted in the West comparing minimally invasive with open gastrectomy for gastric cancer [36, 37]. These developments may aid in diminishing the differences between the East and West.

## Conclusion

Improved outcomes are observed in both Western and Asian studies following minimally invasive gastrectomy in comparison to open procedures. There are differences in patient characteristics between the Western and Asian population. Overall outcomes, such as lymph node yield, complications, and mortality, seem to be in favor of the Asian population. These differences may fade with centralization of care for gastric cancer patients in the West and increasing surgical experience.

## Appendix

**Table:**

Recent queries				
Search	Add to builder	Query	Items found	Time
#5	Add	Search (#4 NOT (“addresses”[Publication Type] OR “biography”[Publication Type] OR “Case Reports” [Publication Type] OR “comment”[Publication Type] OR “directory”[Publication Type] OR “editorial”[Publication Type] OR “festschrift”[Publication Type] OR “interview”[Publication Type] OR “lectures”[Publication Type] OR “legal cases”[Publication Type] OR “legislation”[Publication Type] OR “letter”[Publication Type] OR “news”[Publication Type] OR “newspaper article”[Publication Type] OR “patient education handout”[Publication Type] OR “popular works”[Publication Type] OR “congresses”[Publication Type] OR “consensus development conference”[Publication Type] OR “consensus development conference, nih”[Publication Type] OR “practice guideline”[Publication Type]) NOT (animals[mh] NOT humans[mh)	902	05:45:48
#4	Add	Search (#1 AND #2 AND #3)	1040	05:45:07
#3	Add	Search (“open Surgery”[tiab] OR “open procedure”[tiab] OR “open operation”[tiab] OR laparotomy[tiab] OR laparotomies[tiab] OR open gastrectomy[tiab] OR “open resection”[tiab] OR “open gastric resection”[tiab] OR “open Surgery”[ot] OR “open procedure”[ot] OR “open operation”[ot] OR laparotomy[ot] OR laparotomies[ot] OR “open gastrectomy”[ot] OR “open resection”[ot] OR “open gastric resection”[ot)	59,229	05:44:38
#2	Add	Search (“Surgical Procedures, Minimally Invasive”[Mesh:NoExp] OR “Laparoscopy”[Mesh] OR “Video-Assisted Surgery”[Mesh] OR “gastrectomy”[MeSH] OR Laparoscopy[tiab] OR Laparoscopies[tiab] OR laparoscopic[tiab] OR videolaparoscop*[tiab] OR abdominoscop*[tiab] OR “Video-Assisted”[tiab] OR “Minimal Surgical”[tiab] OR “Minimally Invasive”[tiab] OR “Minimal Invasive”[tiab] OR “Minimal Access Surgical Procedures”[tiab] OR Laparoscopy[ot] OR Laparoscopies[ot] OR laparoscopic[ot] OR videolaparoscop*[ot] OR abdominoscop*[ot] OR “Video-Assisted”[ot] OR “Minimal Surgical”[ot] OR “Minimally Invasive”[ot] OR “Minimally Invasive”[ot] OR “Minimal Access Surgical Procedures”[ot)	189,153	05:44:07
#1	Add	Search (“Stomach Neoplasms”[Mesh] OR Stomach Neoplasm*[tiab] OR Gastric Neoplasm*[tiab] OR Stomach Cancer*[tiab] OR Gastric Cancer*[tiab] OR Stomach carcinoma*[tiab] OR gastric carcinoma*[tiab] OR stomach tumor*[tiab] OR stomach tumour*[tiab] OR gastric tumor*[tiab] OR gastric tumour*[tiab] OR stomach neoplasia*[tiab] OR cardia carcinoma*[tiab] OR linitis plastica[tiab] OR Stomach Neoplasm*[ot] OR Gastric Neoplasm*[ot] OR Stomach Cancer*[ot] OR Gastric Cancer*[ot] OR Stomach carcinoma*[ot] OR gastric carcinoma*[ot] OR stomach tumor*[ot] OR stomach tumour*[ot] OR gastric tumor*[ot] OR gastric tumour*[ot] OR stomach neoplasia*[ot] OR cardia carcinoma*[ot] OR linitis plastica[ot)	99,259	05:43:31
